# Integrating Finite Element Simulation with Actual GTAW Weld Profiles to Optimize Root Height in Stainless Steel 316L Pipe Joints

**DOI:** 10.3390/ma19061088

**Published:** 2026-03-12

**Authors:** Mohammad Sohel, Vishal S. Sharma, Aravinthan Arumugam

**Affiliations:** 1 Engineering Institute of Technology, West Perth, WA 6005, Australia; vishal.sharma@eit.edu.au (V.S.S.);; 2 Engineering Institute of Technology, Melbourne Campus, Melbourne, VIC 3000, Australia

**Keywords:** finite element analysis (FEA), stress concentration factors (SCFs), gas tungsten arc welding (GTAW), load case (LC), the American Society of Mechanical Engineers (ASME), nondestructive testing (NDT)

## Abstract

Weld root reinforcement is a critical geometric parameter governing stress concentration and structural performance in thin-walled stainless-steel piping systems designed to ASME B31.3. While current codes specify permissible dimensional limits, they do not explicitly quantify how incremental variations in root height influence stress distribution under realistic service loading conditions. This study integrates finite element analysis (FEA) with experimentally validated GTAW weld profiles to evaluate the structural influence of weld root height in 316L stainless-steel pipe joints. An experimentally manufactured 4 in schedule 10S joint with a measured root height of less than 1.5 mm was adopted as the baseline geometry. Additional models with reinforcement heights of 1.138, 2.0, 2.5, and 3.0 mm were evaluated under two representative load cases: (i) internal pressure combined with drag and axial thrust (LC-1), and (ii) internal pressure with thrust only (LC-2). The results demonstrate that reinforcement heights exceeding 2.0 mm increase von Mises, hoop, longitudinal, and radial stress gradients, with peak stresses shifting toward the weld toe under drag-inclusive loading. In contrast, reinforcement ≤2 mm provides smoother load transfer and reduced stiffness discontinuity across the weld interface. The combined numerical and experimental findings support a stress-informed upper limit of 2 mm for weld root reinforcement in thin-walled stainless-steel pipelines, offering a performance-based complement to existing dimensional acceptance criteria.

## 1. Introduction

### 1.1. Background

The structural reliability of pipeline systems depends critically on the integrity of girth welds, which act as primary sites of load transfer under internal pressure and service-induced forces. In thin-walled stainless steel piping, even small geometric deviations at the weld, particularly variations in weld root height, can significantly alter local stiffness and increase stress concentration factors (SCFs), which are widely recognized as key drivers of fatigue crack initiation and long-term structural degradation.

Finite element investigations have consistently demonstrated that weld geometry parameters, including reinforcement height, toe radius, and toe angle, significantly influence SCFs under tensile, bending, and shear loading conditions. Quantitatively validated formulations derived from large finite element datasets demonstrate that local stress response is highly sensitive to weld profile geometry rather than nominal stress alone [1]. These findings highlight the importance of geometry-dependent stress assessment when evaluating welded joints in thin material.

In practical operation, pipelines are rarely subjected to internal pressure alone. Additional actions, such as axial thrust and flow-induced effects, are often represented in structural models through superimposed axial and bending loads, which interact with weld geometry and alter both the magnitude and location of peak stresses near the weld root and toe. Modern structural stress formulations employed in finite element post-processing provide a mesh-insensitive framework for linking local stress states to fatigue-relevant behaviors in welded joints, reinforcing the need for stress-based assessment of reinforcement features rather than purely dimensional evaluation [2].

Recent numerical studies on pipeline girth welds incorporating combined internal pressure and bending moments have demonstrated that superimposed loading elevates critical stress measures and can shift stress hot spots toward the weld toe region. These results underscore the importance of evaluating conservative service load envelopes instead of pressure-only conditions when assessing welded joint performance in piping systems [3].

### 1.2. Literature Review

Geometric features of butt and girth welds, particularly root height, flank angle, and toe radius, are well established as primary drivers of local stress concentration and, consequently, fatigue crack initiation near the weld toe and weld root. Parametric finite element investigations on butt-welded plates subjected to tensile, bending, and shear loads have quantified how weld geometry governs stress concentration factors (SCFs), providing validated datasets for geometry-sensitive assessment of welded joints [1,4,5]. Complementary comparative studies and fatigue-based evaluations further confirm that simplified or idealized weld shapes can under-predict local hot spot stresses when reinforcement geometry is not represented realistically in numerical models [6,7].

Because welded joints are commonly evaluated using stress-based integrity and durability criteria, considerable effort has been devoted to defining stress measures that are robust and mesh-insensitive in finite element post-processing. The structural stress method introduced by Dong [2] enables separation of membrane and bending stress components at welded joints and has been widely adopted to improve the consistency of fatigue-relevant stress extraction. Subsequent investigations comparing hot-spot and structural stress procedures demonstrate that the selected stress extraction methodology can significantly influence computed peak stresses and the identification of failure-critical locations [8,9,10]. These frameworks are particularly relevant for girth welds, where steep stress gradients exist across the weld base metal transition and are sensitive to the as-welded profile.

For pipeline systems, internal pressure alone rarely represents the full-service environment. In practice, girth welds are subjected to superimposed axial forces and bending effects arising from thermal expansion, system constraints, weight, and flow-related actions, which can be represented in structural models through combined pressure–axial–bending load envelopes. Numerical studies on girth-welded pipes subjected to combined internal pressure and bending moments show that critical stress measures and fracture-driving forces can increase markedly and that the location of maximum stress may shift between the weld root and toe depending on loading interactions [3,11,12]. These findings reinforce the need to assess weld geometry under conservative multi-action loading scenarios rather than relying solely on pressure-only conditions.

Fabrication imperfections further interact with root geometry to influence stress concentration, particularly axial and angular misalignment. Finite element investigations on girth-welded pipes have demonstrated that misalignment can significantly elevate local stress concentration factors and alter the distribution of peak stresses at the weld root and toe regions, even under moderate loading conditions [13]. Lotsberg analytically examined stress concentration effects caused by axial misalignment at butt welds in pipelines, demonstrating that geometric discontinuities can amplify local stresses relative to nominal axial loading [14,15]. More recent parametric and experimental studies indicate that the combined effects of weld geometry and misalignment must be considered simultaneously, as changes in root profile or assembly tolerance can introduce competing influences on SCF and fatigue performance [16,17]. These findings support the use of as-built weld geometry, rather than idealized profiles, in structural integrity assessments.

From a modeling perspective, girth weld performance is also influenced by welding-induced residual stresses and distortion, which modify mean stress levels and local constraint conditions. Detailed numerical investigations of multi-pass girth welds have demonstrated that both axisymmetric and three-dimensional modeling approaches can capture characteristic residual stress distributions in stainless steel pipe joints [18,19]. Further finite element studies focusing on welding process parameters in 316L stainless steel pipes show that heat input, welding sequence, and thermal boundary conditions significantly affect axial and circumferential residual stress fields, highlighting the importance of process-consistent modeling inputs when linking simulation results to practical fabrication conditions [20]. In addition, numerical experimental investigations of girth-welded slender pipes reveal that global deformation modes, including bending deformation, can be significant and may require mitigation measures, again emphasizing that service-relevant loading and realistic geometry should be assessed together [21].

Overall, prior work establishes that (i) weld reinforcement and root geometry are first-order drivers of SCF and hot-spot stress [1,4,5]; (ii) stress extraction methodology in finite element post-processing influences predicted peak stresses at welded discontinuities [2,8,9,10]; (iii) combined loading involving pressure and axial/bending effects elevates and redistributes critical stress hot spots [3,11,12,22]; and (iv) misalignment and as-built geometric variations materially affect fatigue-relevant stress states and should not be neglected in integrity qualification [13,14,15,16,17]. In addition to numerical investigations, full-scale experimental testing has been essential in validating girth weld behavior under strain-based and complex loading conditions. Large-scale strain tests highlight the sensitivity of weld performance to geometry and deformation capacity [23], while subsequent work refined safety factor calibration within strain-based engineering critical assessment (ECA) frameworks [24]. More recently, full-scale fatigue and burst tests on notched girth welds confirmed that geometric discontinuities significantly influence crack initiation and failure behavior under multiaxial loading [25].

These findings directly motivate the present study’s approach of integrating measured GTAW weld root profiles into an axisymmetric finite element framework and evaluating stress response under two service-relevant load envelopes to identify a root height range that minimizes stress concentration while remaining practical for fabrication and inspection.

### 1.3. Problem Statement

Current acceptance limits for weld root reinforcement height in thin-walled stainless steel piping are predominantly based on empirical geometric criteria specified in design and fabrication codes. While such limits provide practical fabrication guidance, they do not explicitly quantify how incremental variations in weld root height influence local stress distribution at the weld root, toe, and weld base metal transition under realistic service loading conditions. As demonstrated in prior numerical and experimental studies, weld geometry is a first-order driver of stress concentration, and small changes in reinforcement profile can lead to disproportionate changes in local stress response.

In many existing assessments, welded pipe joints are evaluated primarily under internal pressure loading. However, in practical operation, thin-walled piping systems are subjected to additional actions such as axial thrust, system restraint, and flow-related effects that introduce bending and secondary stresses. Neglecting these combined loading effects can result in non-conservative stress predictions and overlook critical stress peaks that govern structural integrity and fatigue susceptibility. Moreover, the interaction between weld root geometry and thin-wall behaviors further amplifies sensitivity to loading mode, making pressure-only evaluation insufficient for reliable integrity assessment.

Despite extensive research on stress concentration factors in welded joints, limited attention has been given to systematic, stress-based evaluation of actual, experimentally achievable GTAW weld root profiles in thin-walled stainless steel pipes under service-relevant combined loading scenarios. In particular, there is a lack of structured comparison that isolates the influence of weld root height across multiple load cases using a consistent pipe geometry and material system. This gap hinders the development of performance-based acceptance criteria that link weld geometry directly to structural stress response rather than relying solely on dimensional limits.

### 1.4. Research Gap

Local stress behaviors in welded joints are governed primarily by geometric discontinuities and loading complexity rather than internal pressure alone [2,6,7,13]. Existing studies consistently demonstrate that weld geometry plays a dominant role in controlling stress concentration and fatigue-relevant stress parameters. However, many numerical investigations continue to rely on idealized weld profiles and simplified loading assumptions, which may obscure the true severity of localized stress amplification when welded joints are subjected to combined axial, bending, or secondary operational loads [6,13].

Thin-walled stainless steel pipelines are particularly sensitive to geometric variations, where relatively small changes in weld root height can produce disproportionately large stress gradients at critical locations such as the weld root, toe, and weld base metal transition [7,13]. Despite this sensitivity, there remains a lack of systematic investigation that isolates the influence of weld root height using realistic, fabrication-achievable weld geometries evaluated under service-relevant combined loading conditions, while maintaining a consistent pipe geometry and material system.

Furthermore, finite element studies of girth-welded pipes frequently lack direct linkage to experimentally validated weld profiles. Numerical stress predictions are seldom correlated with macrostructural examination of physically produced welds, limiting confidence in the applicability of simulation-based conclusions to practical welding outcomes. Consequently, a unified, stress-based assessment framework that integrates finite element simulation with experimentally validated GTAW weld root geometry is still required to establish performance-based acceptance criteria for thin-walled stainless steel pipeline girth welds.

### 1.5. Scope of the Present Study

This study examines the structural influence of weld root height in thin-walled stainless steel piping joints through finite element analysis (FEA). While current ASME-based codes permit a range of root reinforcement heights based primarily on dimensional acceptance criteria, such geometric variations can significantly influence local stress distribution and long-term structural performance.

To address this issue, numerical models were developed for weld root heights of 1.138 mm, 2.0 mm, 2.5 mm, and 3.0 mm, representing profiles commonly observed in gas tungsten arc-welded (GTAW) stainless steel pipe joints. The structural response of each configuration was evaluated under two service-relevant loading scenarios: Load Case 1 (design pressure combined with drag and thrust loads) and Load Case 2 (design pressure combined with thrust only). These load cases reflect realistic operating conditions in thin-walled process piping systems where internal pressure, axial force, and flow-induced effects coexist.

This scope establishes the basis for a systematic assessment of how weld root geometry governs stress concentration behaviors and provides the foundation for developing stress-informed acceptance criteria for stainless steel pipeline girth welds.

### 1.6. Objective

This study aims to address the identified research gap through the following objectives:

1. Develop an axisymmetric, linear elastic finite element (FEA) model of a 4 in schedule 10S stainless steel pipe girth weld incorporating four representative weld root heights (1.138, 2.0, 2.5, and 3.0 mm).
2. Quantify the resulting stress responses (von Mises, hoop, longitudinal, and radial stresses) under two service-relevant load scenarios:
  (1) LC-1 (design internal pressure + drag pressure + axial thrust) and
  (2) LC-2 (design internal pressure + axial thrust).
3. Evaluate the influence of weld root height on the development and location of stress concentration zones at the weld root, weld toe, and across the weld–base metal transition.
4. Correlate finite element stress predictions with macrostructural examination of GTAW girth welds produced through welding parameter optimization to validate the stress-based trends identified in the numerical analysis.
5. Propose a performance-based weld root height range that minimizes stress concentration while maintaining adequate root height and overall weld integrity for thin-walled stainless steel piping systems.

## 2. Materials and Methods

### 2.1. Model Inputs and Assumptions

The finite element model was developed for a 4 in schedule 10S stainless steel pipeline, consistent with the geometry and operating conditions of the company system under study. The design pressure of 35 bar and flow rate of 35 ton/h were taken directly from the current company process data to ensure operational relevance. Material properties were defined as isotropic, linear-elastic stainless steel with an elastic modulus of 2.0 × 10^5^ MPa, Poisson’s ratio of 0.3, and density of 882.1 kg/cm^3^.

**Figure 1:**
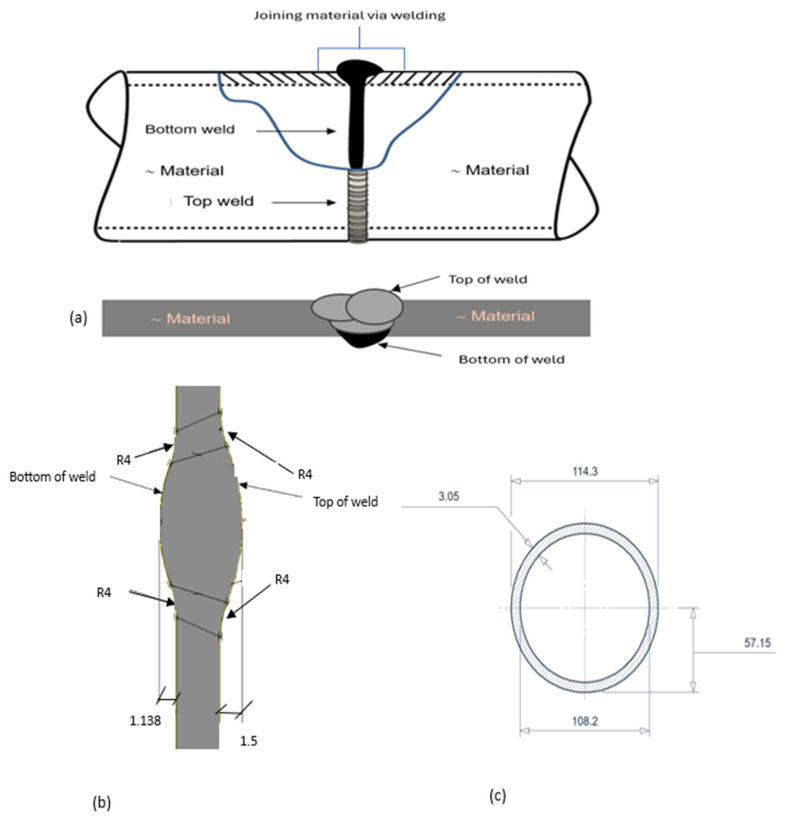
Weld and pipe geometry for finite element modeling of the SS316L pipe joint: (**a**) weld root location; (**b**) parametric weld cross-sectional area; (**c**) nominal pipe geometry (OD, ID, wall thickness). All dimensions are in mm.

The weld geometry implemented in the finite element model is presented in Figure 1. A schematic illustration showing the physical location of the internal weld root relative to the pipe wall thickness (top and bottom of the weld) is presented in Figure 1a. The parametric weld profiles adopted for the FEA simulations are shown in Figure 1b, where four internal root reinforcement heights (1.138, 2.0, 2.5, and 3.0 mm) were analyzed while maintaining a constant weld root radius of 4 mm. The nominal geometry of the 4 in schedule 10S pipe, including the outer diameter, inner diameter, and wall thickness, is shown in Figure 1c.

In addition to chemical composition verification, the mechanical properties of the base material were obtained from the certified Mill Test Certificate and validated against the relevant ASTM [26]/ASME [27] material specifications. The base metal exhibited a yield strength of 277 MPa and an ultimate tensile strength of 593 MPa, confirming compliance with the specified grade requirements.

Similarly, the filler metal used in this study was Avesta GT 316L filler wire (manufactured by Outokumpu Stainless AB, Avesta, Sweden). The filler metal classification and its corresponding mechanical properties were identified in accordance with the applicable filler metal standard to ensure metallurgical compatibility with the base metal and full compliance with the governing welding code requirements.

Weld and pipe geometry definition for finite element modeling of the SS316L pipe joint: (a) parametric weld cross-section with scaled internal root reinforcement heights, (b) nominal pipe geometry showing outer diameter (OD), inner diameter (ID), and wall thickness, (c) actual internal weld root profile, and (d) schematic identification of the weld root relative to the pipe wall thickness.

**Figure 2:**
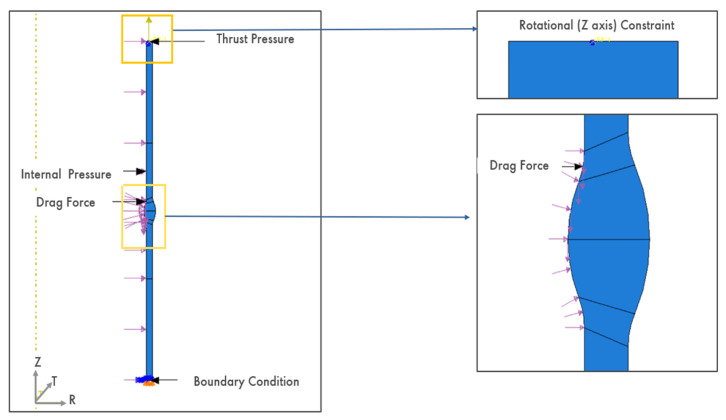
Boundary conditions and applied loads in the finite element model. LC-1 includes internal pressure, end-cap thrust, and drag force, whereas LC-2 includes internal pressure and thrust only. Arrows denote the direction of applied loads, and the highlighted region indicates the weld root location.

Two load cases were applied: LC-1 combined internal pressure, drag-induced shear, and end-cap thrust, while LC-2 included only internal pressure and thrust, as shown in Figure 2.

### 2.2. Assumption and Scope

An axisymmetric, linear static analysis was adopted to isolate geometric influences on stress distribution. Residual stresses, plasticity, and thermal distortions were not considered; the model represents post-fabrication weld geometry. Mesh refinement was concentrated at the weld root and toe, with convergence checks ensuring accuracy of stress concentration factors. These assumptions allowed the study to focus specifically on the structural implications of weld root reinforcement height under realistic operating conditions. This study only considers the geometry of the weld after the fabrication process and does not consider residual stress caused by distortion due to welding. Linear static axisymmetric analysis is performed.

### 2.3. Practical Significance and Contribution

The significance of this analysis is its contribution to a stress-based understanding of weld root reinforcement height in thin-walled stainless steel pipelines. Existing design codes provide empirical limits on reinforcement height, but they do not explicitly quantify the interaction between root geometry and combined loading conditions. By applying finite element analysis to systematically evaluate multiple root reinforcement heights under realistic operational scenarios, this work provides evidence-based guidance that complements current dimensional criteria.

The findings offer practical value for welding engineers, inspectors, and design teams by establishing reinforcement thresholds that balance root height integrity with minimized stress concentration. In doing so, the study advances performance-based acceptance approaches that can enhance fatigue resistance, improve the reliability of welded joints, and support safer long-term operation of stainless steel piping systems.

### 2.4. Overview of the Research Design

This study employs a finite element analysis (FEA) framework to quantify the influence of weld root height on the stress distribution of a 4 in schedule 10S stainless steel pipe girth weld. A linear static axisymmetric model was developed to capture the elastic stress response under two representative operational scenarios:

Load Case 1 (LC-1): Design pressure combined with drag pressure and thrust force.

Load Case 2 (LC-2): Design pressure combined with thrust force only.

By comparing these two cases, the analysis distinguishes between conservative (LC-1) and simplified (LC-2) conditions, providing insight into how reinforcement height alters stress concentration zones under different service environments.

### 2.5. Geometry Assumptions

The pipe was modeled as a 4 in schedule 10S stainless steel pipe with an outside diameter of 114.3 mm and a nominal wall thickness of 3.05 mm. The joint was defined as a square butt weld with a 45° groove angle, reflecting common GTAW practice for thin-walled stainless steel piping.

Key assumptions include:

- Axisymmetric representation of the weld, reducing computational cost while retaining accuracy.
- Post-fabrication weld geometry was modeled, excluding residual stresses and thermally driven plastic distortions.
- The groove angle and cross-sectional weld profile parameters were maintained constant across all models to isolate the influence of root reinforcement height. Only the reinforcement height was varied parametrically, while other geometric features remained unchanged.

The inclusion of residual stresses would require a coupled thermo-mechanical welding simulation that incorporates heat input and thermo-plastic strain evolution, which is beyond the scope of this geometry-focused investigation.

Deng and Kiyoshima [28] employed thermo-mechanical finite element modeling to predict residual stress distributions in a SUS304 girth-welded pipe, with particular emphasis on stress concentration near the weld start and end locations. Their study demonstrated how transient thermal gradients and subsequent cooling generate non-uniform axial and circumferential residual stress fields in welded pipe structures. Perić et al. [29] combined numerical simulation with experimental measurements to evaluate residual stresses in a thick-walled buried-arc welded pipe, highlighting the influence of welding parameters, boundary conditions, and thermal–plastic interaction on the final stress state.

These studies confirm that welding-induced residual stresses arise from localized heating and constrained cooling during fabrication and may significantly modify the pre-service stress condition of welded pipes. However, because the present study focuses on the comparative geometric influence of weld reinforcement under service loading, the exclusion of residual stresses does not affect the relative assessment of reinforcement height effects.

### 2.6. Weld Geometry and Root Heights

Four reinforcement heights were evaluated: 1.138 mm, 2.0 mm, 2.5 mm, and 3.0 mm. A weld root radius of 4 mm was maintained for consistency across all models. These dimensions reflect ranges typically observed in GTAW joints for stainless steel pipelines.

Cross-sectional profiles were digitized and scaled proportionally to ensure realistic weld bead transitions. This approach allowed accurate representation of the reinforcement geometries while maintaining geometric consistency.

### 2.7. Finite Element Model Setup

Simulations were performed in Abaqus/Standard 2023 (Dassault Systèmes, Providence, RI, USA) using the following setup:

- Element type: 2D axisymmetric quadrilateral (CAX4)
- Local mesh refinement was applied at the weld root and toe regions, where the highest stress gradients occur. Mesh adequacy was assessed through progressive local refinement to ensure stable stress contour distribution and consistent peak stress locations across all investigated reinforcement configurations. Further densification of the critical regions did not produce any significant change in peak stress location or overall stress trends. Therefore, the adopted mesh was considered sufficient to capture the relative influence of weld root height on stress redistribution for comparative evaluation within the linear-elastic axisymmetric framework of the present study.
- Model extent: Ten times the wall thickness on either side of the weld, reducing boundary influence on local stress fields.

The axisymmetric formulation was defined in the R–Z coordinate system. The symmetry axis was constrained in the radial direction, and additional displacement constraints were applied to eliminate rigid-body motion while permitting axial deformation under the applied load cases.

### 2.8. Loading and Boundary Conditions

Two load cases were implemented:

- LC-1: Internal design pressure of 35 bar applied to the pipe bore, end-cap thrust applied as uniform end pressure, and a distributed drag pressure representing flow-induced shear.
- LC-2: Internal design pressure of 35 bar and thrust loading only, without drag.

The drag component in LC-1 was estimated using turbulent flow correlations for a mass flow rate of 35 tons/h, with a fluid density of 882.1 kg/m^3^.

#### Drag Load Formulation

The drag load was estimated based on the dynamic pressure associated with internal turbulent flow. The mass flow rate of 35 tons/h corresponds to:

$$\dot{m}=9.722\;\mathrm{kg}/s$$

With a fluid density of:

$$\rho =882.1\;{\mathrm{kg}/m}^{3}$$

The volumetric flow rate is:

$$Q=\frac{\dot{m}}{\rho }=0.01102\;m^{3}/s$$

For an internal diameter of 0.1082 m, the cross-sectional area is:

$$A=0.00919\;m^{2}$$

Yielding a mean flow velocity of:

$$V=\frac{Q}{A}=1.20\;m/s$$

The drag pressure was approximated using the dynamic pressure expression:

$$p_{D}=\frac{1}{2}\rho V^{2}=635\;\mathrm{Pa}\;(0.000635\;\mathrm{MPa})$$

Compared to the internal design pressure of 35 bar (3.5 MPa), the drag component is negligible (<0.02%) and therefore does not significantly influence the peak stress magnitude. It is retained in Load Case 1 to represent combined pressure–flow interaction within the present geometry-focused investigation.

## 3. Results

### 3.1. Load Case 1 (Design Pressure + Drag Pressure + Thrust Force)

#### 3.1.1. Von Mises Stress Distribution Under Load Case 1 (Design Pressure + Drag Load)

**Figure 3:**
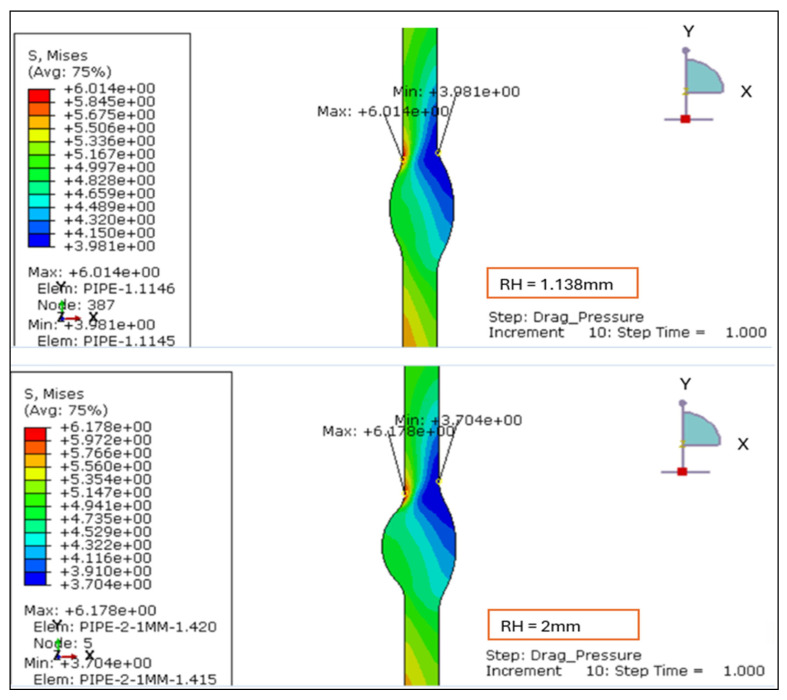
Von Mises stress plots: LC-1: Drag + Design for root height 1.138 mm and 2 mm.

**Figure 4:**
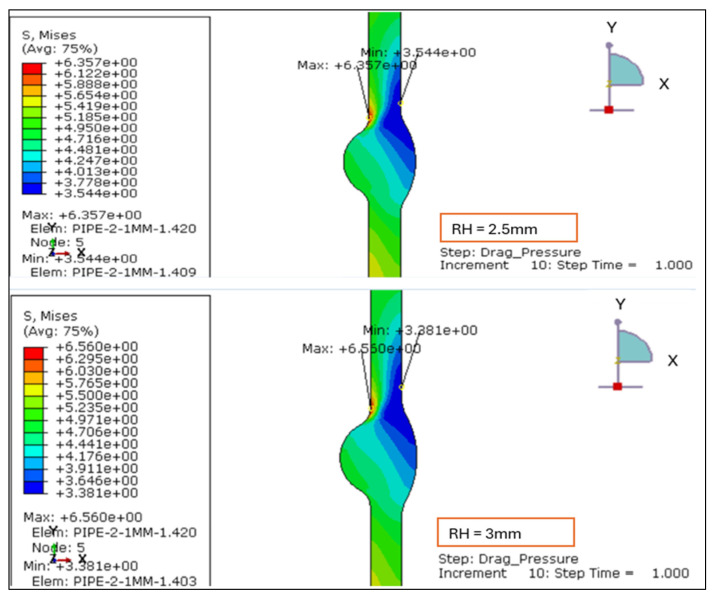
Von Mises stress plots: LC-1 Drag + Design for root height 2.5 mm and 3 mm.

The von Mises stress contours for four weld configurations with reinforcement heights of 1.138 mm, 2.0 mm, 2.5 mm, and 3.0 mm were evaluated. The analysis reveals that the magnitude and location of the maximum equivalent stress are strongly influenced by the degree of reinforcement, as illustrated in Figure 3 and Figure 4.

For the smallest reinforcement height of 1.138 mm, the stress field is uniformly distributed across the weld cross-section, with a maximum von Mises stress of approximately 6.0 MPa concentrated near the weld toe. This configuration demonstrates the most favorable behavior, exhibiting limited stress localization and a smooth transition into the adjacent base metal. Increasing the reinforcement to 2.0 mm produces a sharper gradient and a higher stress maximum (6.178 MPa), with the peak region shifting upward toward the external surface.

At 2.5 mm, the maximum von Mises stress further rises (6.357 MPa) and becomes more concentrated at the toe, indicating local stiffening and elevated constraint due to the thicker reinforcement profile. The 3.0 mm case shows a slight redistribution of stresses but maintains a high intensity (6.56 MPa), suggesting that excessive reinforcement does not relieve but instead sustains elevated local stress zones.

Overall, the results confirm that reinforcement heights above 2.0 mm amplify the equivalent stress at critical weld transition regions. The 1.138 mm model yields the lowest stress concentration and the most uniform distribution, confirming it as the optimum configuration under drag-inclusive loading. The behavior under LC-1 highlights that the drag component intensifies near-surface shear and magnifies von Mises stress at the weld toe, thus validating the need to limit reinforcement to ≤2 mm in thin-walled stainless-steel pipelines.

#### 3.1.2. Radial Stress Distribution Under Load Case 1 (Design Pressure + Drag Load)

**Figure 5:**
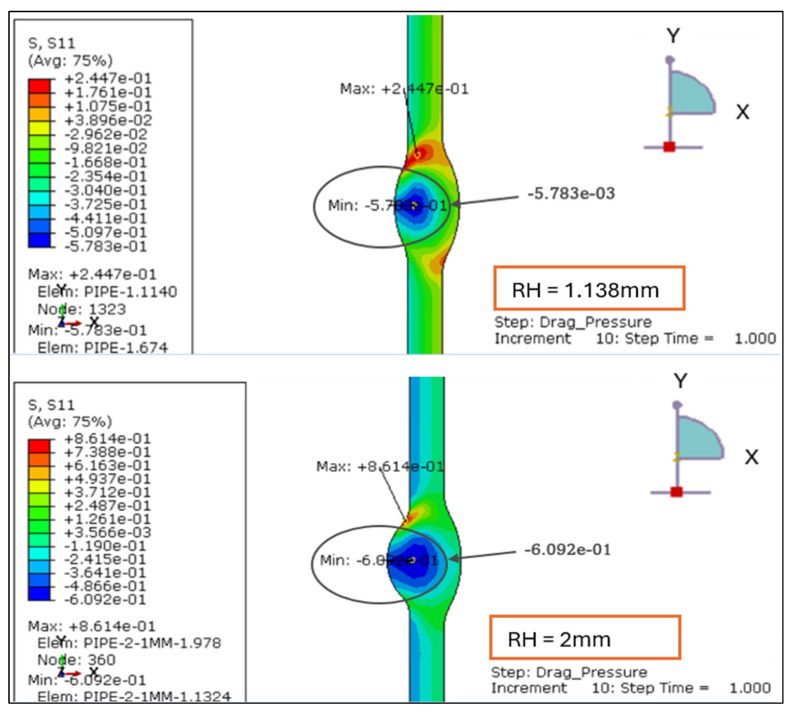
The radial stress results for LC-1 (Drag + Design) for load case 1.138 mm and 2 mm.

**Figure 6:**
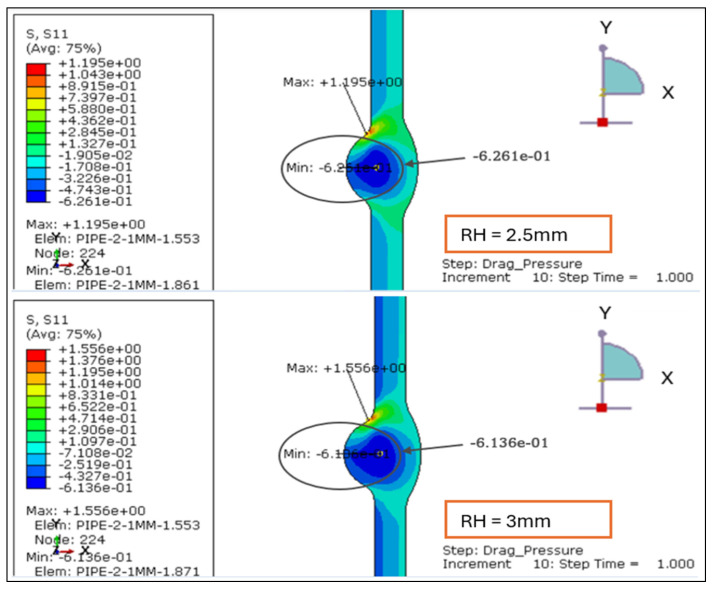
The radial stress results for LC-1 (Drag + Design) for load case 2.5 mm and 3 mm.

The radial stress contours for four root reinforcement heights (1.138 mm, 2.0 mm, 2.5 mm, and 3.0 mm) under the combined effects of internal design pressure and drag load were evaluated. The results demonstrate how increasing weld reinforcement height modifies through-thickness stress gradients and local compressive zones near the weld root, as illustrated in Figure 5 and Figure 6.

For the smallest reinforcement (1.138 mm), the stress field remains almost uniform, exhibiting a minor compressive zone at the weld root (−0.006 MPa) and a moderate tensile response at the inner wall (+0.24 MPa). This configuration provides the most balanced radial behavior, suggesting minimal stiffness discontinuity across the weld-to-base-metal interface.

When reinforcement increases to 2.0 mm, a significant rise in radial compression (−0.61 MPa) is observed near the root, accompanied by higher tensile stress at the inner surface (+0.86 MPa). The greater stiffness of the thicker reinforcement restricts radial deformation and creates a localized constraint. At 2.5 mm, the radial compression deepens slightly (−0.63 MPa), and the tensile region extends to +1.19 MPa, producing a sharper stress gradient across the section. The 3.0 mm profile sustains comparable compression (−0.61 MPa) but yields the highest tensile peak (+1.56 MPa), with a distinct concentration around the weld toe.

Overall, the findings confirm that larger reinforcement heights (>2 mm) increase through-thickness stress gradients and amplify compressive mismatch at the weld interface. Reinforcement ≤ 2 mm maintains a smoother and more uniform radial distribution, indicating a structurally preferable configuration under the combined influence of design pressure and drag loading.

#### 3.1.3. Longitudinal Stress Plot Under Load Case 1 (Design Pressure + Drag Load)

**Figure 7:**
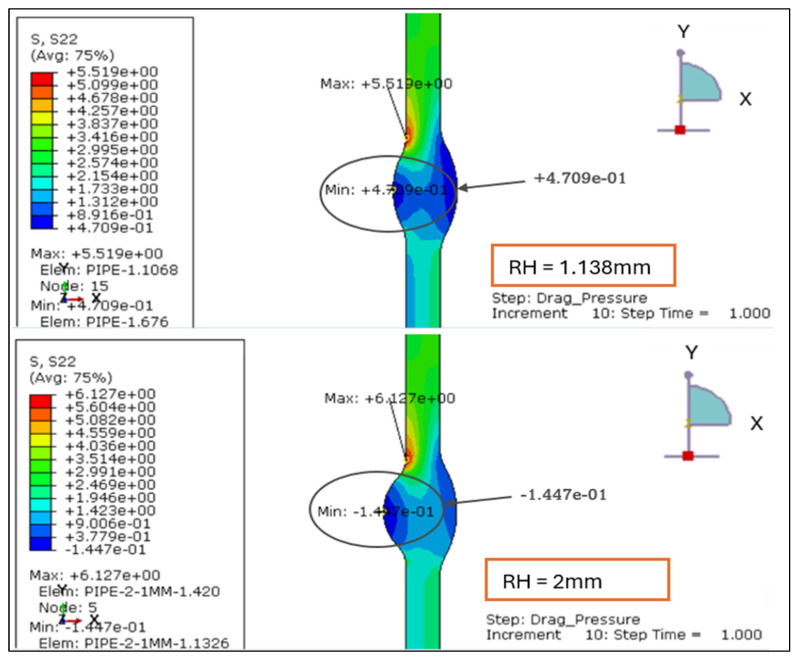
The longitudinal stress results for LC-1 (Drag + Design) for load case 1.138 mm and 2 mm.

**Figure 8:**
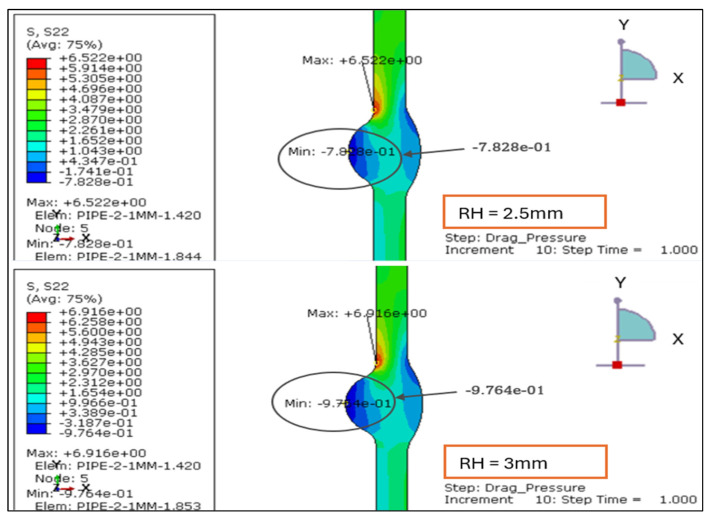
The longitudinal stress results for LC-1 (Drag + Design) for load case 2.5 and 3 mm.

The longitudinal stress contours for root weld reinforcement heights of 1.138, 2.0, 2.5, and 3.0 mm. For the smallest reinforcement (1.138 mm), the entire section remains under tension, with stress ranging from +0.47 MPa to +5.52 MPa. This indicates uniform axial loading and minimal bending influence, as shown in Figure 7 and Figure 8.

At 2.0 mm, a slight compressive region (−0.14 MPa) develops near the weld root while the tensile zone rises to +6.13 MPa, signaling the onset of local bending caused by drag-induced eccentricity. When reinforcement increases to 2.5 mm, tensile stress peaks at +6.52 MPa and compression deepens to −0.78 MPa, showing greater stiffness contrast between the weld crown and base wall.

At 3.0 mm, the tensile maximum reaches +6.92 MPa and compression extends to −0.97 MPa, forming the steepest axial stress gradient and the most localized high-stress region around the weld toe.

Overall, the results demonstrate a clear transition from uniform tension at 1.138 mm to alternating tension–compression patterns beyond 2 mm of reinforcement. This evolution confirms that excessive reinforcement height promotes axial bending and stress reversal under drag-inclusive conditions, whereas reinforcement ≤ 2 mm maintains a balanced longitudinal stress field and improved structural performance.

#### 3.1.4. Hoop Stress Plot Under Load Case 1 (Design Pressure + Drag Load)

**Figure 9:**
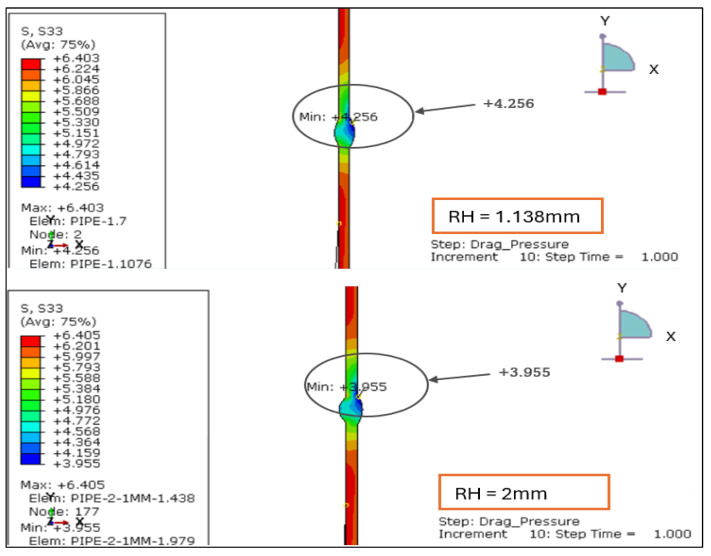
Hoop-stress contours for root height 1.138 mm and 2 mm for LC-1 (Drag + Design).

**Figure 10:**
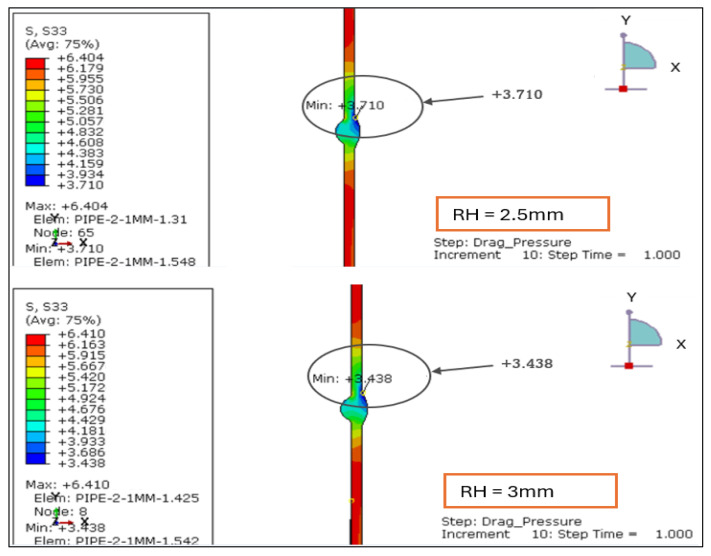
Hoop-stress contours for root height 2.5 mm and 3 mm for LC-1 (Drag + Design).

The hoop-stress contours for root weld reinforcement heights of 1.138, 2.0, 2.5, and 3.0 mm were evaluated. All cases remain tensile through the wall, but the stress uniformity deteriorates as reinforcement increases, as shown in Figure 9 and Figure 10.

- RH = 1.138 mm: The field is comparatively uniform with a modest gradient across the weld region.
- RH = 2.0 mm: A clearer local trough forms at the weld, indicating increased stiffness contrast.
- RH = 2.5 mm: Hoop stress concentrates more strongly near the toe/transition.
- RH = 3.0 mm: This case exhibits the least uniform hoop field and the steepest local gradient.

A useful measure is the hoop range (Δ*σθ* = *σθ*,*max* − *σθ*,*min*), which captures the severity of the local gradient shown below:

○ 1.138 mm: Δ*σθ* = 6.403 − 4.256 = 2.147 MPa
○ 2.0 mm: Δ*σθ* = 6.405 − 3.955 = 2.450 MPa
○ 2.5 mm: Δ*σθ* = 6.404 − 3.710 = 2.694 MPa
○ 3.0 mm: Δ*σθ* = 6.410 − 3.438 = 2.972 MPa

While the peak tensile level (~6.4 MPa) remains nearly constant across models, the minimum hoop stress drops as reinforcement height grows, expanding Δ*σθ* and sharpening the near-toe gradient. Under design + drag loading, this behavior reflects local stiffening of the reinforced profile: drag-induced shear couples with the thicker weld crown, pulling hoop stress toward the toe and reducing the local minimum.

Based on the above analysis, reinforcement > 2.5 mm yields the largest hoop gradient and the most pronounced localization, conditions associated with higher structural hot-spot stress and fatigue risk. Configurations < 2 mm retain higher minima and smaller Δ*σθ*, indicating a more uniform and favorable hoop-stress distribution under LC-1.

#### 3.1.5. Quantitative Stress Concentration Factor (SCF) Assessment (LC1 and LC2)

To provide a quantitative metric for evaluating geometric stress amplification, the Stress Concentration Factor (SCF) was calculated for each reinforcement height. The SCF is defined as:

$$SCF=\frac{\sigma \theta ,max}{\sigma \theta ,nom}$$

where:

- $\sigma_{\theta ,max}$ is the maximum local hoop stress extracted at the weld hot-spot region from the finite element results.
- $\sigma_{\theta ,\mathrm{nom}}$ is the nominal thin-wall membrane hoop stress calculated as:

$$\sigma \theta ,nom=\frac{PD}{2t}$$

Using the following:

Design pressure *P* = 0.35 MPa, internal diameter *D* = 108.2 mm, and wall thickness *t* = 3.05 mm:

$$\sigma \theta ,nom=\frac{0.35\;\times \;108.2}{2\;\times \;3.05}$$

$$\sigma \theta ,nom=\frac{37.87}{6.10}$$

σθ,nom=6.21 MPa

The SCF values under LC-1 (Design Pressure + Drag Load) are calculated in Table 1 below:

The results indicate that the peak-based SCF remains relatively constant (approximately 1.03) across all root heights for both LC-1 and LC-2. This observation confirms that under membrane-dominated pressure loading, the absolute peak hoop stress magnitude is primarily governed by thin-wall pressure theory rather than local geometric variation. In other words, the reinforcement height does not significantly increase the maximum membrane stress level, which remains close to the nominal thin-wall prediction.

However, although the peak-based SCF remains nearly constant, this metric alone does not fully capture the geometric severity introduced by increasing root height. As previously demonstrated through the increasing hoop stress range (Δ*σθ*), root height substantially affects stress redistribution and gradient severity near the weld toe. In thin-walled geometries, stress gradient amplification and local stiffness mismatch are often more sensitive indicators of structural severity than peak stress magnitude alone.

While SCF values remain close to unity, the expanding Δ*σθ* (from 2.147 MPa to 2.972 MPa under LC-1) indicates increasing localization and structural hot-spot intensity with root height ≥ 2.5 mm. A similar trend is observed under LC-2, confirming that the geometric effect primarily manifests through stress gradient amplification rather than peak stress escalation.

Therefore, in thin-walled stainless steel piping subjected primarily to internal pressure, reinforcement height influences structural performance predominantly through stress redistribution and local gradient intensification, rather than through substantial amplification of peak membrane stress.

This distinction is important: while SCF remains close to unity, increasing reinforcement height progressively sharpens local stress gradients at the weld toe and root transition, which are critical regions for fatigue crack initiation and long-term structural degradation.

### 3.2. Load Case 2 (Design Pressure + Thrust Force)

#### 3.2.1. Von Mises Stress Distribution Under Load Case 2 (Design Pressure + Thrust Force)

**Figure 11:**
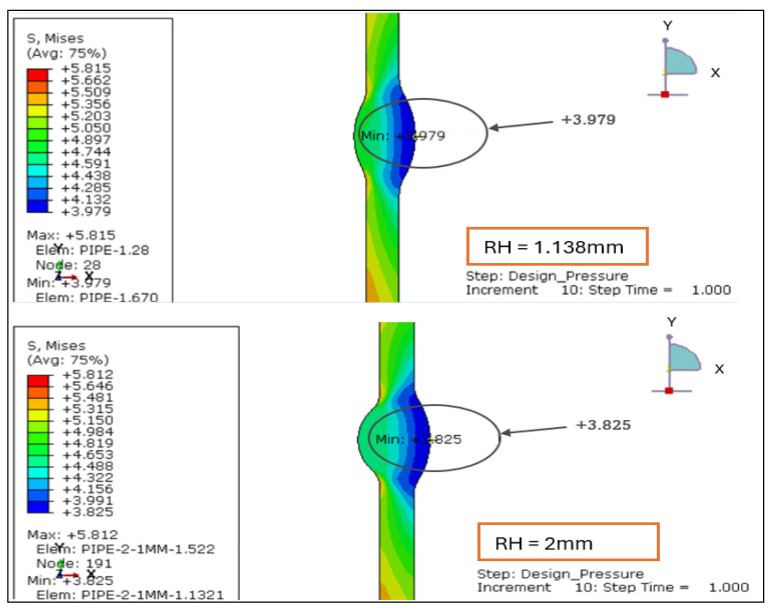
Von Mises Stress Distribution under Load Case 2 (Design Pressure + Thrust Force) for root height 1.138 mm and 2 mm.

**Figure 12:**
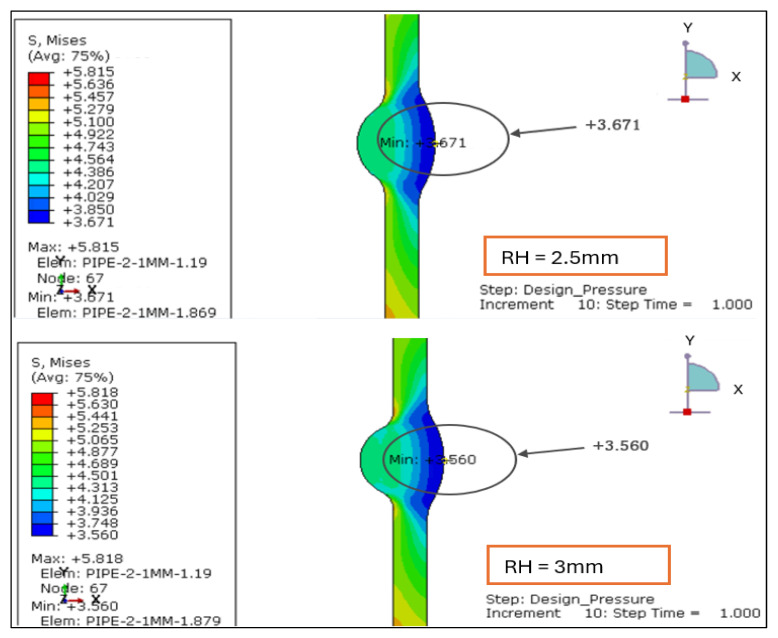
Von Mises Stress Distribution under Load Case 2 (Design Pressure + Thrust Force) for root height 2.5 and 3 mm.

The von Mises equivalent stress contours (S Mises) for reinforcement heights of 1.138, 2.0, 2.5, and 3.0 mm were evaluated under Load Case 2, where only the design pressure and thrust forces were applied. Compared with LC-1, the stress magnitudes are slightly lower because drag-induced shear is absent, allowing a purer pressure-dominated stress response, as shown in Figure 11 and Figure 12.

- The 1.138 mm configuration exhibits a nearly uniform distribution across the weld region, indicating balanced internal pressure distribution with minimal local amplification.
- A mild increase in stress concentration is observed at 2.0 mm reinforcement, particularly near the weld toe, while overall uniformity is largely maintained.
- With reinforcement increasing to 2.5 mm, stress gradients become noticeably steeper along the fusion line, suggesting elevated local stiffness mismatch.
- The 3.0 mm configuration produces the largest stress differential across the weld region, indicating the highest degree of local concentration.

Table 2 below for stress-range (Δ*σv* = *σv*,*max* – *σv*,*min*) values shows that this progressive intensification of local stress differs with reinforcement height. As reinforcement height increases, Δ*σv* rises from 1.84 MPa to 2.26 MPa, showing a clear growth in local stiffness mismatch. The 1.138 mm configuration maintains the most uniform stress field, while reinforcement ≥ 2.5 mm produces a sharper von Mises gradient along the weld toe, where fatigue and crack initiation risks are typically highest.

Overall, the LC-2 results reaffirm that welds with reinforcement < 2 mm provide the most favorable stress profile under pure internal-pressure and thrust loading, whereas larger reinforcements amplify stress concentration without improving structural performance.

#### 3.2.2. Radial Stress Distribution Under Load Case 2 (Design Pressure + Thrust Force)

**Figure 13:**
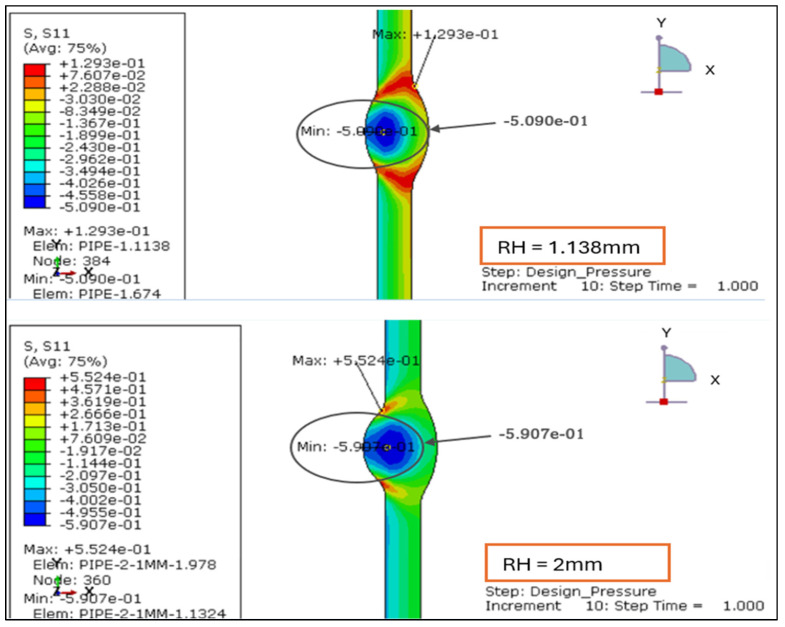
Radial stress distribution under Load Case 2 (Design Pressure + Thrust Force) for root height 1.138 mm and 2 mm.

**Figure 14:**
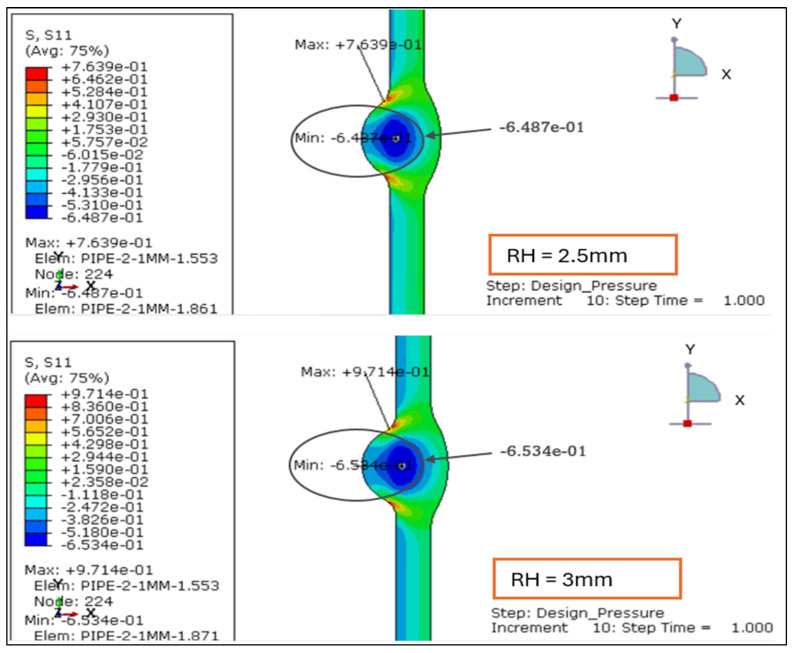
Radial stress distribution under Load Case 2 (Design Pressure + Thrust Force) for root height 2.5 mm and 3 mm.

The radial stress distribution for four reinforcement heights from 1.138 mm, 2.0 mm, 2.5 mm, and 3.0 mm was evaluated under Load Case 2, where the pipe is subjected only to internal design pressure and end thrust. The radial stress remains compressive at the weld root and tensile near the inner wall, consistent with the expected through-thickness stress profile of a thin-walled pressure pipe as shown in Figure 13 and Figure 14.

- RH = 1.138 mm: The radial field is well balanced, showing a mild compressive band at the root and smooth transition across the weld region. Stresses are symmetrically distributed, indicating stable deformation.
- RH = 2.0 mm: Increased reinforcement height strengthens the local stiffness, producing a more distinct compressive zone at the weld interface. The contrast between inner and outer wall stresses widens, marking the onset of localized constraint.
- RH = 2.5 mm: The compressive intensity rises sharply near the root, while tensile stresses develop closer to the pipe’s mid-wall. The stress contour becomes asymmetric, implying a higher stiffness gradient along the weld transition.
- RH = 3.0 mm: This configuration shows the most concentrated stress pattern, with steep gradients across the weld zone and strong confinement of compressive stress at the root.

Table 3 (below) shows the overall stress range (Δ*σr* = *σr*,*max* – *σr*,*min*) and demonstrates how increasing reinforcement amplifies through-thickness stress differences. The progression from 1.138 mm to 3.0 mm reveals a clear amplification of compressive and tensile extremes, increasing Δ*σr* by nearly 10 MPa. This suggests that thicker reinforcements impose stronger geometric constraints on radial deformation, leading to higher local stiffness and stress concentration at the weld root.

Based on the above, reinforcement heights < 2 mm promote smoother radial stress diffusion and lower mismatch, preserving more uniform load transfer through the wall thickness. Consequently, maintaining reinforcement below 2 mm is structurally favorable for thin-walled stainless steel pipes operating under design pressure and thrust forces, mitigating the risk of subsurface cracking and root defect propagation.

#### 3.2.3. Longitudinal Stress Distribution Under Load Case 2 (Design Pressure + Thrust Force)

**Figure 15:**
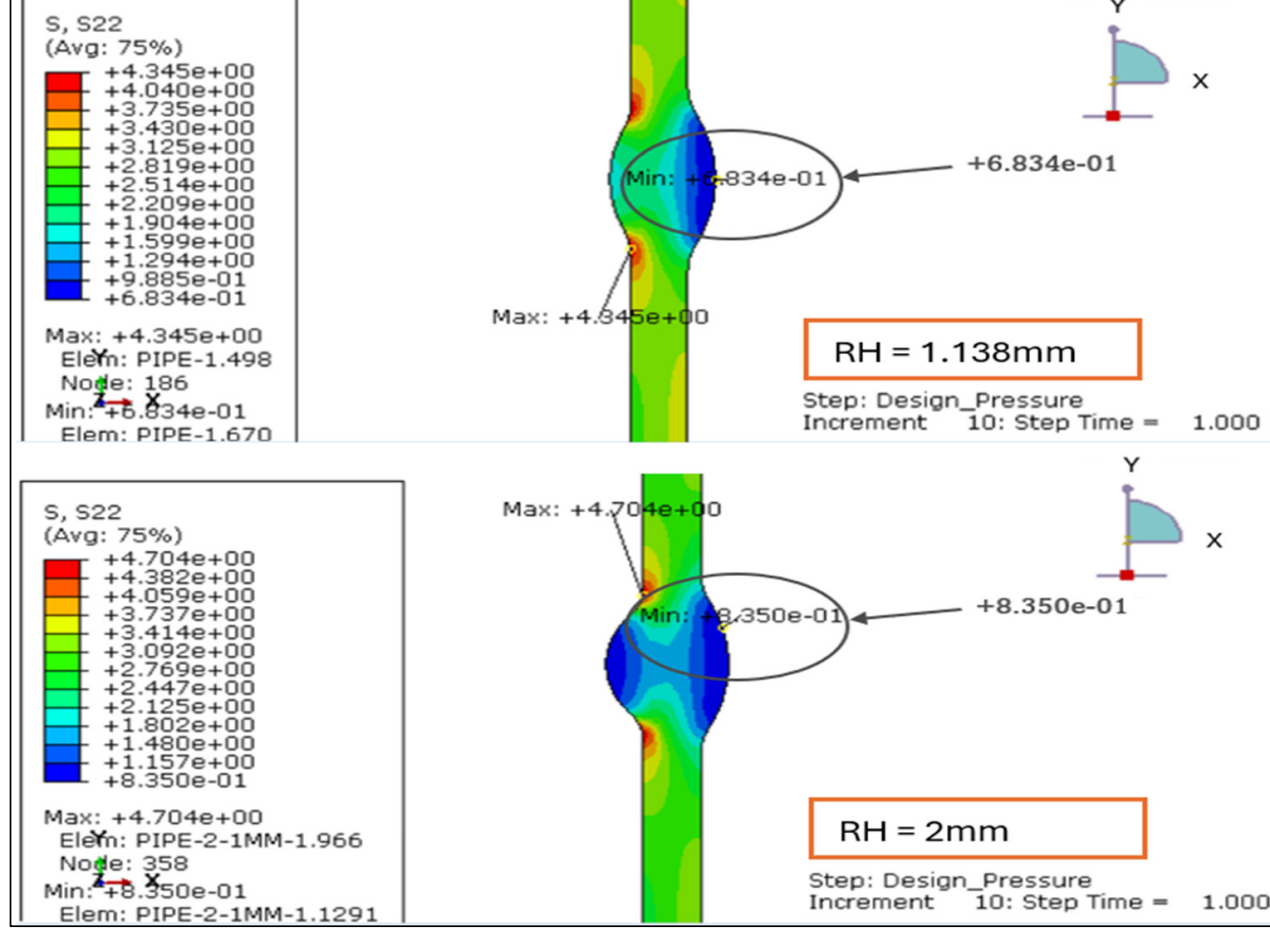
Longitudinal stress plot under Load Case 2 (Design Pressure + Thrust Force) for root height 1.138 mm and 2 mm.

**Figure 16:**
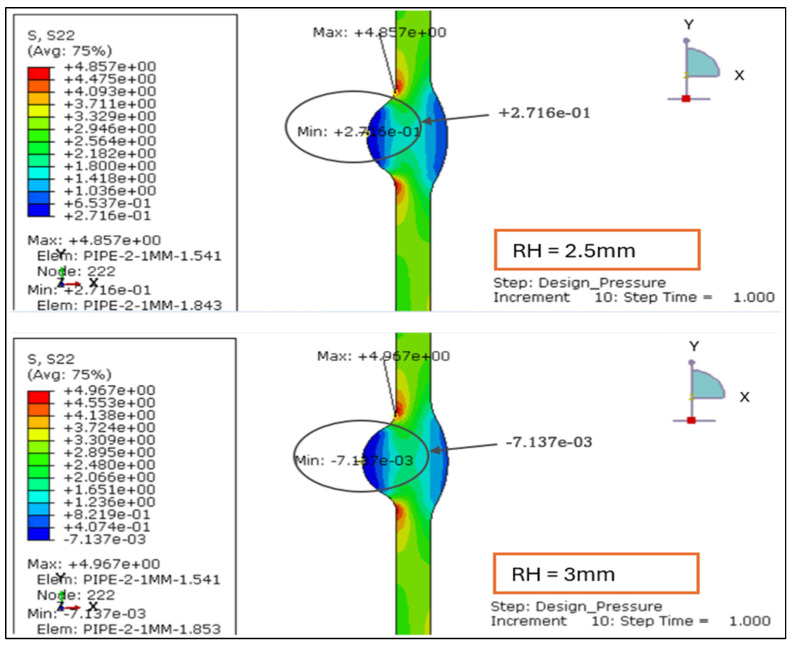
Longitudinal stress plot under Load Case 2 (Design Pressure + Thrust Force) for root height 2.5 mm and 3 mm.

The longitudinal stress distributions for weld reinforcement heights of 1.138 mm, 2.0 mm, 2.5 mm, and 3.0 mm were evaluated under Load Case 2, where the pipe is subjected to internal design pressure and axial thrust only. The results indicate that longitudinal stresses are primarily tensile along the pipe wall but may shift to compressive zones near the weld toe and root, depending on reinforcement geometry, as shown in Figure 15 and Figure 16.

- RH = 1.138 mm: The stress distribution is uniform and symmetric across the weld, showing no compressive reversal. The lower reinforcement height ensures balanced axial load transfer and a smooth stress transition between the weld and base metal.
- RH = 2.0 mm: A mild increase in tensile magnitude is observed, with the gradient concentrated near the weld toe. The section stiffness begins to influence axial load sharing, indicating the onset of localized bending restraint.
- RH = 2.5 mm: The tensile band narrows and shifts toward the weld crown, suggesting partial axial constraint and higher stress localization. This configuration demonstrates greater sensitivity to axial mismatch effects.
- RH = 3.0 mm: The stress contour shows the steepest gradient, with tensile peaks concentrated at the weld toe. Although no compressive reversal occurs, the localized amplification suggests bending-induced stress intensification under thrust loading.

The corresponding longitudinal stress range (Δ*σx* = *σx*,*max* – *σx*,*min*) for each case is summarized in Table 4 below:

The above results confirm that higher reinforcement heights produce greater longitudinal stress variation, reflected by the increasing Δ*σx* from 3.66 MPa to 4.97 MPa. The additional stiffness from excessive reinforcement amplifies tensile stresses at the weld toe, introducing greater axial mismatch between the weld metal and the parent pipe. For thin-walled stainless-steel pipelines, reinforcement heights < 2 mm maintain uniform longitudinal stress distribution and are therefore preferable for improved fatigue resistance and structural stability under design pressure and thrust conditions.

#### 3.2.4. Hoop Stress Distribution Under Load Case 2 (Design Pressure + Thrust Force)

**Figure 17:**
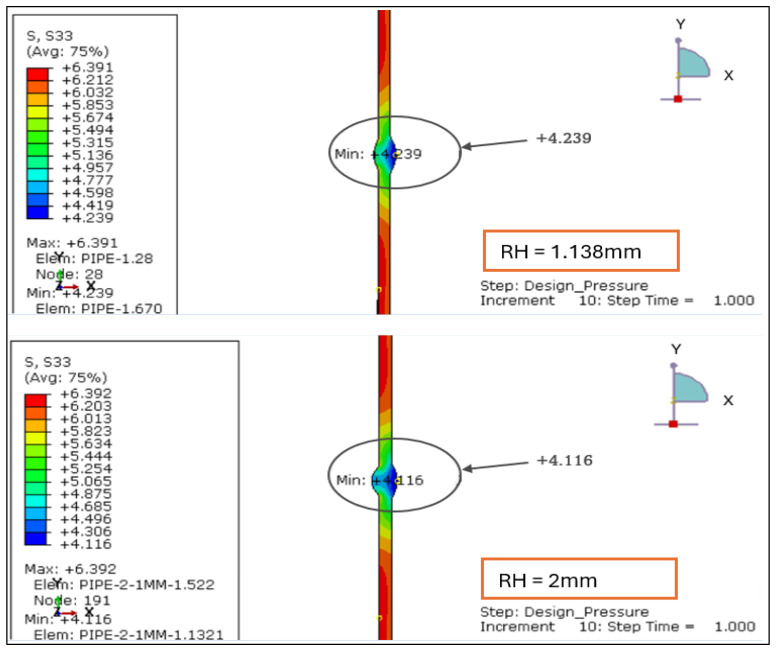
Hoop stress distribution under Load Case 2 (Design Pressure + Thrust Force) for load cases 1.138 mm and 2 mm.

**Figure 18:**
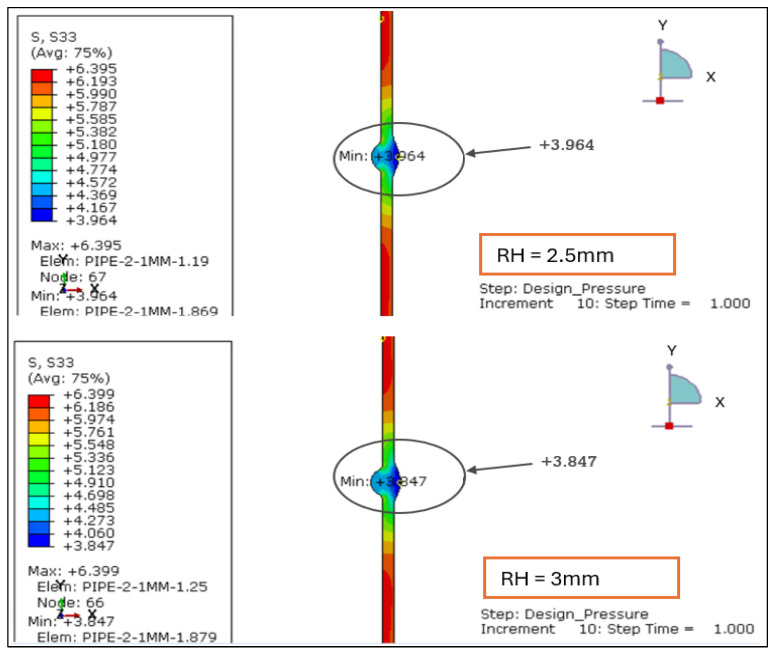
Hoop stress distribution under Load Case 2 (Design Pressure + Thrust Force) for load cases 2.5 mm and 3 mm.

The hoop stress distribution for reinforcement heights of 1.138 mm, 2.0 mm, 2.5 mm, and 3.0 mm was evaluated under Load Case 2, which includes design pressure and thrust force. As expected for thin-walled cylindrical sections, hoop stress remains the dominant component, governing circumferential wall loading and pipe expansion behaviors, shown in Figure 17 and Figure 18.

- RH = 1.138 mm: The stress contours are uniform across the weld and adjacent pipe wall, suggesting stable circumferential stress flow. The relatively small stress range (Δ*σθ* ≈ 2.152 MPa) confirms that this configuration maintains minimal local discontinuity.
- RH = 2.0 mm: A marginal rise in stress range (Δ*σθ* ≈ 2.276 MPa) indicates a slight stiffness increase. The localized concentration begins to emerge near the weld crown, but the stress field remains predominantly tensile and evenly distributed.
- RH = 2.5 mm: The hoop stress minimum decreases as reinforcement height increases, reflecting elevated constraint effects at the toe. This produces a larger stress gradient (Δ*σθ* ≈ 2.431 MPa) and demonstrates that reinforcement beyond 2 mm alters circumferential load uniformity.
- RH = 3.0 mm: The highest reinforcement case exhibits the widest stress spread (Δ*σθ* ≈ 2.552 MPa). Stress concentration becomes visible at both the root and toe regions, suggesting that excessive reinforcement amplifies local bending and ovalization tendencies.

The summary of Hoop Stress Range (Δ*σθ* = *σθ*,*max* – *σθ*,*min*) for each case is summarized in Table 5.

Hoop stress results confirm that circumferential tension increases with reinforcement height, primarily due to the elevated stiffness mismatch between the weld and adjacent pipe wall. The consistent rise in Δ*σθ* from 2.15 MPa to 2.55 MPa demonstrates that reinforcement above 2.0 mm leads to greater stress concentration and reduced structural uniformity under pure pressure and thrust conditions. For thin-walled stainless-steel pipelines, maintaining weld root height at or below 2 mm ensures smoother hoop stress transitions, minimizing fatigue sensitivity and enhancing dimensional stability under service pressure.

### 3.3. Experimental Validation Through Macrostructural Examination

#### Actual Experiment Validation via Marcostructural Examination

**Figure 19:**
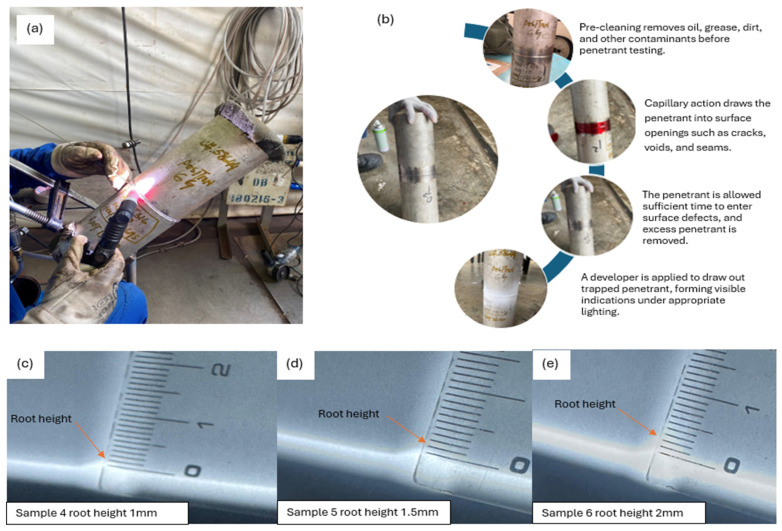
(**a**) GTAW welding of SS316L pipe joint. (**b**) Liquid penetrant testing steps. (**c**) Root height 1.0 mm. (**d**) Root height 1.5 mm. (**e**) Root height 2.0 mm.

**Figure 20:**
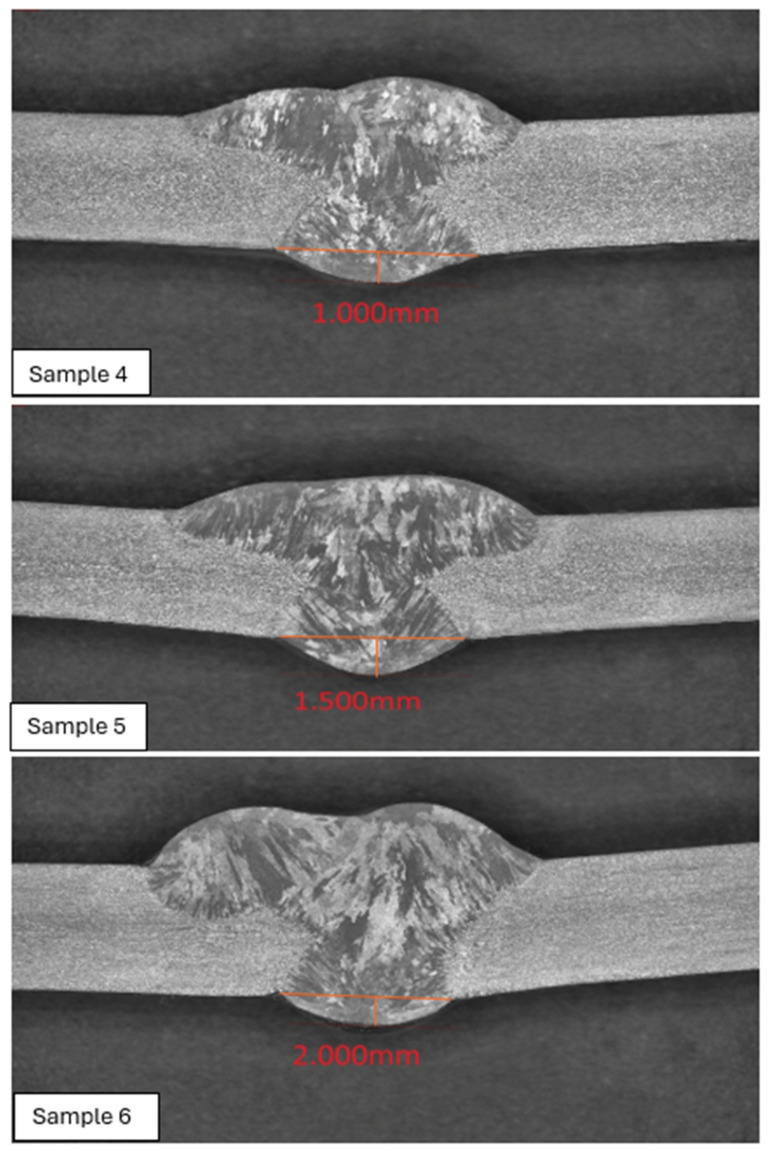
Macrostructural examination of GTAW weld joints produced under optimized parameter combinations, illustrating controlled root penetration heights used for experimental validation of FEA-predicted stress behavior.

In parallel with the finite element simulations, a total of nine GTAW specimens were fabricated using different parameter optimization sets to produce controlled variations in root penetration height. Multiple GTAW parameter combinations were systematically optimized to generate weld joints with controlled and reproducible root heights. Following welding, all specimens underwent visual inspection, after which three samples (4, 5, and 6) were identified as visually acceptable, as shown in Table 6. These samples were subsequently subjected to liquid penetrant testing (PT) and radiographic testing (RT) to confirm that they were free from surface and internal defects before macrostructural examination, as shown in Figure 19. The macrostructural examination confirms that the achieved root penetration heights (approximately 1.0 mm, 1.5 mm, and 2.0 mm) closely align with the root height range investigated in the numerical model, as shown in Figure 20.

Root height measurements were obtained from all macro-examined specimens, and the observed values showed limited variability within each parameter group, indicating acceptable geometric consistency for comparison with the finite element model. Notably, the macrograph corresponding to Sample 5, with an average root height of approximately 1.5 mm, exhibits the most uniform penetration profile and balanced bead geometry. This experimentally observed weld morphology is consistent with the FEA results, which indicate a stable and uniformly distributed stress field with minimal stress concentration for root heights below or equal to 2 mm under both loading cases.

Conversely, samples exhibiting either reduced or increased penetration width show greater geometric variability at the weld root, which corresponds to the stress gradients and localized stress amplification predicted by the finite element analysis for higher root heights. The experimental results, therefore, provide direct physical validation of the FEA-based stress trends and support the identification of an optimal weld root height window for stainless steel piping joints.

## 4. Discussion

### 4.1. Comparative Behavior of LC-1 and LC-2

Across all root height cases, LC-1 generated higher stresses than LC-2. The penalty was most severe in the 2.0–2.5 mm configurations, where drag significantly elevated von Mises, hoop, and longitudinal stresses. Radial stress also became more compressive under LC-1, particularly at larger reinforcement heights.

By contrast, welds with root height ≤ 2 mm reinforcement demonstrated smaller LC-1 vs. LC-2 differentials, preserving a safer operational envelope. Practical Implication: Designing and qualifying the weld root at ≤2 mm under LC-1 inherently guarantees safe performance under LC-2.

### 4.2. Narrative Synthesis

The dominant mechanisms below explain why reinforcement < 2 mm consistently outperforms larger values:

- Geometry–Stiffness Coupling: Larger reinforcement increases local stiffness, concentrating stresses at the weld toe. The 2.0–2.5 mm range exhibited the most severe concentration, whereas 1.138 mm remained within a smoother stress envelope.
- Drag Amplification: LC-1 magnified stress gradients by introducing surface shear. This effect penalized higher reinforcement, thereby widening the gap between LC-1 and LC-2 for cases with 2.0–2.5 mm.
- Axial Eccentricity: Larger protrusions produced higher longitudinal tensile stresses, particularly under LC-1. Reinforcement ≤ 2 mm avoided this penalty.
- Through-Thickness Uniformity: Radial stress profiles were more uniform below 2 mm, reducing mismatch at the root interface and limiting cyclic damage initiation.

In thin-walled piping systems, membrane stress dominates the absolute stress magnitude under internal pressure loading. Consequently, geometric variations such as weld root reinforcement height influence structural response more prominently through stress redistribution and local gradient amplification rather than through substantial increases in peak membrane stress. This explains why the calculated SCF values remain close to unity across all configurations, while stress ranges (Δ*σ*) and local gradients increase systematically with reinforcement height.

Table 7 below summarizes the maximum and minimum stress components extracted from the weld root region for all investigated weld root heights under both loading cases. The reported stress includes von Mises equivalent stress as well as hoop, longitudinal, and radial stress components, enabling a comprehensive assessment of stress magnitude, distribution, and directional dominance as a function of weld root geometry.

Based on the comparative Table 6 above, it is evident that both load cases exhibit consistent stress trends across the analysed weld root heights, though with distinct differences in magnitude and localization. The inclusion of drag loading in LC-1 (Design + Drag + Thrust) leads to a systematic rise in all stress components, such as von Mises, hoop, longitudinal, and radial, compared to LC-2 (Design + Thrust), confirming that drag loading amplifies near-surface shear and bending effects.

At the smallest root height of 1.138 mm, the stress field remains uniform, with balanced hoop and longitudinal distributions and minimal radial distortion. This configuration demonstrates smooth load transfer between the weld and base metal, yielding the most stable response to stress under both load conditions.

As root height increases to 2.0 mm, a moderate rise in hoop and longitudinal stresses is observed, indicating the onset of localized stiffness variation; however, the overall response remains within acceptable limits, suggesting that this root height still provides safe performance for thin-walled stainless-steel joints.

Beyond root height 2.5 mm, a noticeable shift in stress concentration occurs. The von Mises and hoop stress maxima increase sharply, with peaks migrating toward the weld toe, an area commonly associated with fatigue crack initiation. The localized stiffness mismatch between the weld crown and pipe wall becomes more pronounced, leading to higher longitudinal gradients and elevated radial compression. Under LC-1, these effects are particularly amplified, confirming that drag forces disproportionately penalize excessive reinforcement geometry.

The root height 3.0 mm case consistently produces the highest stress differentials and most severe localization, characterized by sharp gradients at the weld toe and root. This condition represents a mechanically inefficient geometry, introducing unnecessary stress amplification that may compromise long-term fatigue resistance.

This narrative and comparative analysis demonstrate that LC-1 produces higher and more critical stress magnitudes than LC-2, underscoring the importance of incorporating drag effects in weld design assessments. Root height of ≤2 mm yields the most favorable combination of low stress concentration, uniform distribution, and minimal radial distortion, while reinforcement ≥ 2.5 mm consistently increases the structural penalty without added benefit. These analyses reinforced that a 2 mm maximum root height should serve as a stress-based acceptance limit for thin-walled stainless-steel pipeline welds operating under combined internal pressure, thrust, and flow drag conditions.

### 4.3. Longitudinal Stress Behavior of LC-1 and LC-2

The variation of longitudinal stress *σx* with increasing weld root height for both load cases is LC1 (Design Pressure + Drag + Thrust) and LC2 (Design Pressure + Thrust). The results demonstrate a clear positive correlation between root height and longitudinal stress magnitude for both load conditions, reflecting the stiffening effect of increased weld root geometry on the axial load path. At the smallest reinforcement, 1.138 mm, the longitudinal stress remains low and evenly distributed, registering approximately 5.519 MPa for LC-1 and 4.345 MPa for LC2. This configuration exhibits the balanced stress sharing between the weld and base metal, confirming that minimal reinforcement promotes smoother axial stress transfer. As root height increases to 2.0 mm, longitudinal stress rises moderately to 6.127 MPa (LC1) and 4.704 MPa for (LC2), signaling the beginning of local stiffness accumulation near the weld toe.

**Figure 21:**
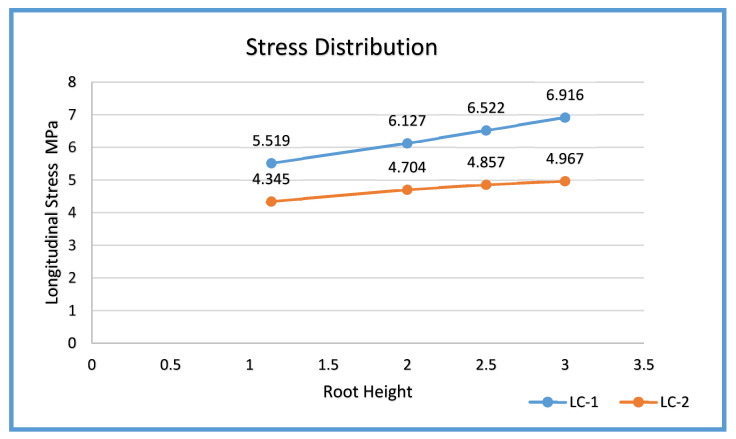
Variation of longitudinal stress *σx* with increasing weld root height for both load cases.

While the weld root height increases to 2.5 mm, results in a sharper divergence between the two cases, where LC1 records 6.522 MPa compared to LC2 at 4.857 MPa. This difference highlights the additional penalty imposed by the drag component in LC1, which elevates axial bending and causes stress amplification near the weld transition. At the maximum root height of 3.0 mm, LC1 reaches 6.916 MPa, while LC2 remains comparatively lower at 4.967 MPa. The widening gap between the two curves (≈2 MPa difference) reinforces the observation that drag effects in LC1 exacerbate longitudinal stress, particularly at excessive reinforcement heights, as shown in Figure 21.

In general, both curves exhibit a monotonic increase in stress magnitude with root height, yet LC1 consistently yields higher stresses across all configurations. The nonlinear slope of LC1 suggests greater sensitivity to geometric variation under combined loading. These findings imply that welds exceeding 2.0 mm reinforcement height introduce undesirable stiffness gradients, amplifying axial stresses that can accelerate fatigue initiation and toe cracking. Conversely, reinforcement heights ≤ 2.0 mm maintain a near-linear and moderate stress profile under both load conditions, confirming their efficiency and compliance with safe design limits.

### 4.4. Statement of Results for the FEA

The FEA outcomes confirm that for 4 in schedule 10S stainless steel girth welds, root heights below 2 mm, particularly the 1.138 mm case, consistently produced the lowest or most uniform stress results across von Mises, hoop, longitudinal, and radial distributions. The benefit was most critical under LC-1, which represents the conservative, drag-inclusive loading scenario. For thin-walled stainless steel piping of this type, reinforcement height should be controled to <2 mm to minimize stress intensification while preserving adequate weld root height.

The stress field analysis demonstrated clear and consistent trends. Von Mises stresses were lowest at 1.138 mm and reached maximum intensity between 2.0 and 2.5 mm, indicating that intermediate root height produces the most severe stress concentrations. Hoop stress, the dominant component in thin-walled geometry, was similarly sensitive, with pronounced peaks at 2.0–2.5 mm. Longitudinal stress transitioned from compressive at 1.138 mm to tensile at reinforcements greater than 2.0 mm, especially under LC-1 conditions, raising fatigue concerns. Radial stresses were compressive in all cases, but gradients became much steeper with reinforcement heights above 2 mm, while remaining smoother and more uniform when reinforcement was ≤2 mm.

### 4.5. Safe vs. Unsafe Configurations

The synthesis of stress responses across all components allows reinforcement heights to be clearly classified as safe or unsafe. Reinforcement ≤ 2 mm, and particularly the 1.138 mm geometry, consistently provided the lowest von Mises and hoop stresses, maintained compressive or neutral longitudinal stresses, and achieved uniform radial fields. These configurations satisfied LC-1, the most conservative load case, and by extension satisfied LC-2.

By contrast, reinforcement ≥ 2.5 mm proved unsafe. These geometries generated peak hoop and von Mises stresses, introduced tensile longitudinal stresses at the weld toe, and produced steep radial gradients. Although such welds may satisfy dimensional code limits, they exceed stress-based safety margins and increase susceptibility to fatigue cracking and defect propagation.

### 4.6. Implications for Welding Codes and Industry Practice

The discussion highlights several practical implications for industry:

- Performance-based acceptance, like code limits, should be supplemented by stress-based criteria, particularly for thin-walled stainless steels operating under combined loading.
- Inspection prioritization on NDT should target welds with reinforcement > 2 mm, as these are more likely to harbor stress concentrations and fatigue hot spots.
- Fabrication control on welders should be trained to achieve reinforcement ≤ 2 mm, supported by quality control programs emphasizing geometry measurement and process discipline.
- Design safety on asset integrity teams should adopt LC-1 style evaluations for pipelines subject to high drag, ensuring that conservative envelopes are satisfied.

### 4.7. Practical Implications for Welding Practice

For welders and inspectors, the results provide a clear target. The root height in thin-walled stainless steel piping should be controlled to ≤2 mm. Although current codes allow larger reinforcement, this research demonstrates that welds with greater root height exhibit increased local stress concentration and steeper stress gradients under elastic service loading, despite being geometrically acceptable.

Reinforcement measurement should therefore be emphasized during both visual inspection and non-destructive testing, and become a key quality-control parameter in acceptance decisions when stress-based performance criteria are considered.

### 4.8. For Piping Design and Asset Integrity Management

From a design perspective, engineers should account for root height when evaluating geometry-driven stress redistribution and structural stress severity under service loading. This consideration is particularly important in pipelines exposed to high flow velocities and turbulence, where combined pressure–flow interactions may contribute to stress redistribution. Reinforcement greater than 2 mm should trigger a fitness-for-service assessment even if dimensional tolerances are formally satisfied, particularly in applications where stress concentration is a governing design factor.

### 4.9. For Welding Standards and Codes

Current standards such as ASME [30], API 1104 [31], and ISO 5817 [32] specify reinforcement height primarily in dimensional terms.

According to ASME B31.3 (Table 341.3.2), for piping with nominal wall thickness less than 6 mm, the maximum allowable internal reinforcement (root height) is 1.5 mm, whereas for thicknesses greater than 6 mm, the allowable limit increases to 3 mm. The schedule 10S pipe investigated in this study has a nominal wall thickness of 3.05 mm; therefore, the applicable ASME limit is 1.5 mm. The experimentally achieved root heights (≤1.5 mm) are fully compliant with this requirement.

In the pipeline sector, the EPRG (European Pipeline Research Group) guidelines on the assessment of defects in transmission pipeline girth welds adopt an engineering critical assessment (ECA)-based framework, emphasizing strain capacity, defect tolerance, and performance under combined loading conditions [33].

In the present study, performance-based acceptance refers to the minimization of geometry-induced stress concentration under service-relevant loading conditions. The primary performance indicators evaluated include peak von Mises stress, hoop stress gradient (Δ*σθ*), and the calculated Stress Concentration Factor (SCF). These quantifiable structural metrics provide engineering-based indicators of weld severity and potential fatigue susceptibility in thin-walled stainless steel piping systems.

This study shows that dimensional limits alone do not capture the structural risks associated with stress concentration. A shift toward performance-based acceptance is warranted, where computational stress evaluation or equivalent analytical approaches supplement geometric checks. In thin-walled stainless steel systems, the results support an upper limit of ≤2 mm as a stress-justified criterion.

While the ASME code ensures minimum dimensional compliance, the present results indicate that stress redistribution becomes progressively more severe for reinforcement heights ≥ 2.5 mm. Therefore, although ≤1.5 mm satisfies the code requirement for the investigated thickness, the structural analysis suggests that maintaining reinforcement within the ≤2 mm range provides improved stress uniformity and reduced gradient severity under combined service loading.

### 4.10. Recommendations

The outcomes of this study lead to a few recommendations. First, root height should be limited to ≤2 mm in thin-walled stainless steel pipelines, with 1.138 mm providing the most favorable distribution of stresses. Second, structural evaluations should be based on LC-1, since it captures the conservative effects of drag and thrust, ensuring that welds are robust against the most demanding conditions. Third, inspection protocols should be revised so that nondestructive testing personnel treat root height measurement as a critical acceptance step. Fourth, welding standards should evolve toward hybrid acceptance criteria that combine geometric and stress-based evaluations. Fifth, fatigue and defect tolerance considerations must be explicitly linked to root height, as welds with reinforcement above 2 mm are particularly vulnerable to crack initiation. Finally, digital weld assessment tools, including FEA, should be integrated into design and inspection workflows to validate weld geometry under realistic operating loads.

### 4.11. Limitations

This FEA analysis assumed idealized, defect-free geometry and material properties and did not account for welding residual stresses or thermal distortion. The weld profile was modeled without geometric defects such as lack of fusion, porosity, or root misalignment in order to isolate the specific structural influence of weld root reinforcement height. Only axisymmetric girth welds were modeled, whereas in practice, misalignment, undercut, and surface irregularities are often present. Moreover, fatigue life predictions were inferred from stress behavior rather than calculated directly using fracture mechanics models or experimental data. The presence of actual welding imperfections in service may further amplify local stress concentration beyond the values predicted in the present idealized model. These limitations suggest that the results, while robust in comparative terms, are conservative rather than absolute predictions of weld performance.

## 5. Conclusions

This FEA study demonstrates that weld root height is not merely a cosmetic or workmanship-related feature, but a critical structural parameter governing the stress response of thin-walled stainless steel pipeline girth welds. Finite element analysis reveals that increasing root height beyond 2 mm leads to a pronounced amplification of von Mises, hoop, and longitudinal stresses, particularly under realistic service conditions that include drag loading. In contrast, maintaining the weld root height below 2 mm, ideally close to the experimentally measured value of approximately 1.138 mm, results in more uniform stress distributions, reduced stress gradients at the weld root, and a more favorable balance between longitudinal and radial stress components.

The numerical findings are strongly supported by experimental macrostructural examination of GTAW welds produced under optimized process parameters. Weld joints achieving root penetration within the 1.0 to 2.0 mm range exhibit uniform fusion boundaries, balanced bead geometry, and consistent penetration profiles, directly corresponding to the low-stress configurations predicted by the finite element models. Microstructural observations further confirm the sound integrity of the weld metal and heat-affected zone, with no evidence of metallurgical discontinuities that would contradict the stress-based performance trends identified through simulation. Together, these experimental results provide physical validation of the FEA-predicted optimal root height window.

Among the investigated loading scenarios, Load Case 1, incorporating internal pressure, axial thrust, and drag effects, represents the most demanding operational envelop and should therefore be adopted as the reference basis for weld qualification and structural assessment. The combined numerical and experimental evidence supports the adoption of a weld root height of ≤2 mm as a performance-based acceptance criterion for thin-walled stainless steel piping systems.

By integrating stress-based finite element evaluation with experimentally validated weld geometry and microstructural integrity, this study demonstrates that a relatively modest geometric control can deliver substantial improvements in structural robustness, defect tolerance, and service reliability. The overarching recommendation is that welding practice, inspection criteria, and future code development should move beyond purely dimensional limits and incorporate stress-informed acceptance criteria to enhance the safety and longevity of critical welded piping systems.

## Figures and Tables

**Table 1:** The stress concentration factor for both load cases 1 and 2.

**Root Height (mm)**	***σv*,*max* (MPa)**	***σv*,*nom* (MPa)**	**SCF (LC-1)**
1.138	6.403	6.21	1.031
2	6.405	6.21	1.032
2.5	6.404	6.21	1.031
3	6.410	6.21	1.032
**Root Height (mm)**	***σv*,*max* (MPa)**	***σv*,*nom* (MPa)**	**SCF (LC-2)**
1.138	6.391	6.21	1.029
2	6.392	6.21	1.029
2.5	6.395	6.21	1.030
3	6.399	6.21	1.031

**Table 2:** The stress-range values of local stress differences with root height.

Root Height (mm)	*σv*,*max* (MPa)	*σv*,*min* (MPa)	Δ*σv* (MPa)
1.138	5.815	3.979	1.836
2	5.812	3.825	1.987
2.5	5.815	3.671	2.144
3	5.818	3.56	2.258

**Table 3:** The overall stress range demonstrates that Δ*σr* increases progressively from 6.383 MPa to 16.248 MPa with reinforcement height.

Root Height (mm)	*σr*,*max* (MPa)	*σr*,*min* (MPa)	Δ*σr* (MPa)
1.138	1.293	−5.090	6.383
2	5.524	−5.907	11.431
2.5	7.639	−6.487	14.126
3	9.714	−6.534	16.248

**Table 4:** Corresponding longitudinal stress range for each root height.

Root Height (mm)	*σx*,*max* (MPa)	*σx*,*min* (MPa)	Δ*σx* (MPa)
1.138	4.345	0.683	3.662
2	4.74	0.835	3.905
2.5	4.857	0.272	4.585
3	4.977	0.007	4.97

**Table 5:** Corresponding hoop stress range for each root height.

Root Height (mm)	*σθ*,*max* (MPa)	*σθ*,*min* (MPa)	Δ*σθ* (MPa)
1.138	6.391	4.239	2.152
2	6.392	4.116	2.276
2.5	6.395	3.964	2.431
3	6.399	3.847	2.552

**Table 6:** Welding parameters and test results for all samples.

Samples	Current (A)	Voltage (V)	Travel Speed (mm/min)	Root Gap (mm)	Visual Inspection (VI)	Actual Root Height (mm)	Radiographic Testing (RT)
1	85	10	50	1	Fail	−1.0 mm	Fail
2	85	11	60	1.5	Fail	−0.5 mm	Fail
3	85	12	70	2	Pass	0.5 mm	Fail
4	90	10	60	1	Pass	1.0 mm	Pass
5	90	11	70	1.5	Pass	1.5 mm	Pass
6	90	12	50	2	Pass	2.0 mm	Pass
7	100	10	70	1	Pass	1.0 mm	Fail
8	100	11	50	1.5	Pass	1.5 mm	Fail
9	100	12	60	2	Pass	1.5 mm	Fail

Note: VI = visual inspection; Pass = Acceptable; Fail = Not Acceptable; RT = Radiography.

**Table 7:** Summary of maximum and minimum von Mises, hoop, longitudinal, and radial stresses at the weld root region for different weld root heights.

		Von Mises *σv* (MPa)		Hoop *σθ* (MPa)		Longitudinal *σx* (MPa)		Radial *σr* (MPa)		Behaviors
Root Height (mm)	Load Case	Max	Min	Max	Min	Max	Min	Max	Min	
1.138	LC-1	6.01	3.98	6.4	4.26	5.52	4.71	0.24	−0.005	It shows a uniform stress field, smooth distribution, minimal concentration (Safe)
	LC-2	5.82	3.98	6.39	4.24	4.35	0.68	0.13	−5.09	Stable stress pattern, dominated by hoop tension, safe & conservative
2	LC-1	6.17	3.7	6.41	3.95	6.13	1.45	0.86	−0.61	Slight rise in SCF, mild axial bending (Acceptable)
	LC-2	5.81	3.83	6.39	4.12	4.7	0.83	0.55	−5.91	Balanced field, moderate stress spread (Acceptable)
2.5	LC-1	6.56	3.54	6.4	3.71	6.52	0.78	1.19	−0.62	Peak stress shifts to the weld toe; the highest SCF (Critical zone begins)
	LC-2	5.82	3.67	6.39	3.96	4.86	0.27	0.76	−6.49	Increased stiffness mismatch, tensile dominance (Moderate risk)
3	LC-1	6.56	3.28	6.41	3.44	6.92	0.97	1.55	−0.61	Localized hot-spot, high stress gradient at toe (Unsafe/overstressed)
	LC-2	5.82	3.56	6.4	3.85	4.96	0.01	0.97	−6.53	Pronounced concentration; stiffness-induced amplification (Unsafe)

## Data Availability

The original contributions presented in this study are included in the article. Further inquiries can be directed to the corresponding author.
